# Network Pharmacology-Based Strategy for Predicting Active Ingredients and Potential Targets of *Coptis chinensis* Franchin Polycystic Ovary Syndrome

**DOI:** 10.1155/2021/6651307

**Published:** 2021-08-18

**Authors:** Jian Xiong Ma, Miaoyong Ye, Ke Ma, Kang Zhou, Yingying Zhang, Xiting Wang, Hongxuan Tong

**Affiliations:** ^1^The Second Clinical Medical College, Zhejiang Chinese Medical University, Hangzhou 310053, China; ^2^Department of Andrology, Dongzhimen Hospital, Beijing University of Chinese Medicine, Beijing 100007, China; ^3^Department of Urology, The Affiliated Wenling Hospital of Wenzhou Medical University, Wenling, Zhejiang 317500, China; ^4^School of Traditional Chinese Medicine, Beijing University of Chinese Medicine, Beijing 100029, China; ^5^Institute of Basic Theory for Chinese Medicine, China Academy of Chinese Medical Sciences, Beijing 100700, China

## Abstract

**Background:**

Polycystic ovary syndrome (PCOS) causes low fertility in females. *Coptis chinensis* (*C. chinensis*) is used to clear heat and dampness, purify fire, and detoxify in traditional Chinese medicine (TCM). Although *C. chinensis* has demonstrated efficacy against PCOS in clinical practice, there are no available data regarding the bioactive components of *C. chinensis*, their targets, and molecular mechanisms underlying their effects.

**Methods and Results:**

Network pharmacology was used to analyze the bioactive components of *C. chinensis*, their targets, and signaling pathways underlying their effects. The TCM systems pharmacology database and analysis platform (TCMSP) was used to screen 14 effective active ingredients and 218 targets of *C. chinensis*. The GeneCards, OMIM, and PharmGkb databases were used to screen 3517 disease targets for PCOS, and 102 common targets of drugs and diseases were screened using R Cytoscape that was utilized to build a drug-active ingredient-disease target interaction network, and the STRING platform was utilized to construct a common target protein-protein interaction network, including 102 nodes and 221 edges. Key targets of *C. chinensis* for the treatment of PCOS included *JUN*, *MAPK*, *IL6*, *CXCL8*, *FOS*, and *IL1B*. A total of 123 gene ontology (GO) terms and 129 pathways were acquired by GO and KEGG enrichment analyses. The AGEs/RAGE, TNF, IL-17, MAPK, and HIF-1 signaling pathways were closely related to PCOS and may be the core pathways involved in PCOS. Schrodinger software was used to evaluate the interaction between active components and their targets and explore binding modes. Furthermore, based on the prediction of network pharmacology study, a mouse model of PCOS was established to evaluate the curative role and underlying mechanisms of *C. chinensis*. The results showed that *C. chinensis* treatment reversed histopathological damage of the ovary and also ameliorated the mRNA and protein expression levels of the predicted hub targets (MAPK1, CXCL8, IL-6, and IL-1*β*). These results indicated that WZYZP has a protective effect on spermatogenesis disorder, suggesting that it could be an alternative choice for male infertility therapy.

**Conclusions:**

This preliminary study verified the basic pharmacological effects and mechanisms of *C. chinensis*, a TCM, in the treatment of PCOS. These results indicate that the therapeutic effects of *C. chinensis* on PCOS may be achieved by regulating the expression of inflammatory factors. This study provides new insights for the systematic exploration of the mechanism of traditional Chinese medicine.

## 1. Introduction

Polycystic ovarian syndrome (PCOS) is an endocrine syndrome characterized by persistent anovulation, hyperandrogenemia, or insulin resistance and is the most common cause of infertility in women of childbearing age [1]. In recent years, PCOS has been defined as a metabolic syndrome, because it is often accompanied by metabolic disorders such as obesity, insulin resistance, hyperlipidemia, and long-term risk of type 2 diabetes, hypertension, and other cardiovascular diseases, seriously affecting the physical and mental health of women of childbearing age [2, 3]. At present, the treatment for insulin resistance in PCOS patients is mainly to increase insulin sensitivity in peripheral tissues, and the representative drug is metformin. Although metformin can obtain a certain effect temporarily, it has many side effects, it cannot be used for a long time, and the recurrence rate after stopping the drug is high [4]. Therefore, it is important to find a PCOS replacement or adjuvant therapy with less side effects and effective results.

In China, *Coptis chinensis* (*C. chinensis*) is a commonly used botanical medicine with a long history and is widely used to treat various diseases, including gynecological diseases and diabetes. In the theory of Chinese medicine, *C. chinensis* can clear away heat and dampness, purify fire, and detoxify the body. Pharmacological studies have confirmed that *C. chinensis* has anti-inflammatory, antiallergenic, hypoglycemic, lipid-lowering, and antioxidant properties [5, 6]. Of note, it can improve insulin resistance and impaired glucose tolerance in PCOS patients [7, 8]. However, based on the multitarget and multipath pharmacological characteristics of Chinese herbal medicines, the current research of *C. chinensis* in the treatment of PCOS still has shortcomings such as low sensitivity, single reliable evaluation index, and inability to systematically evaluate it. It is difficult to make a scientific, effective, and comprehensive explanation of its mechanism of action. Therefore, it is important to systematically and comprehensively study the material basis and mechanisms of action of *C. chinensis* to improve and treat PCOS.

Network pharmacology is based on the theory of systems biology. A model is established for predicting the relationship between drugs and targets, diseases and therapeutic targets, integrating the network of interactions between them, and analyzing the interaction of active ingredients and targets in the network module. It is a systematic and holistic approach to the study of the relationships between active pharmaceutical ingredients and potential targets [9, 10]. It can scientifically and comprehensively evaluate the potential active ingredients, targets, and mechanisms of action of traditional Chinese medicine (TCM) and traditional Chinese medicinal compounds [11].

**Figure 1:**
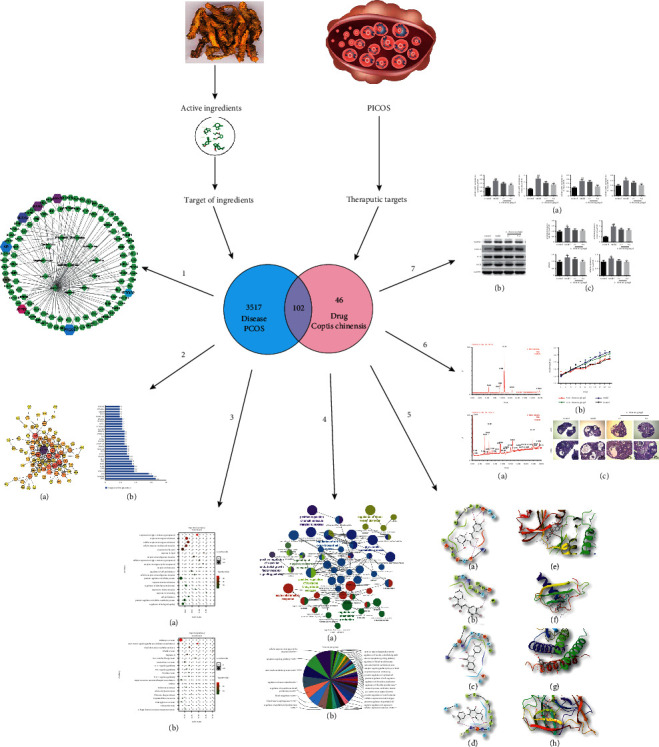
Flow chart depicting the network pharmacology approach used in this study.

In this study, a network pharmacology method was used to analyze the active ingredients of a TCM (*C. chinensis*) and explore its possible targets and mechanisms of action in treating PCOS. Further, an experiment model was established to evaluate the effect of *C. chinensis* on PCOS. The flow chart of the current study is shown in Figure 1.

## 2. Materials and Methods

### 2.1. Research Material

The following databases and resources were used: Chinese Herbal Medicine System Pharmacology Platform (TCMSP) database (https://lsp.nwu.edu.cn/tcmsp.php); Uniprot database (https://www.uniprot.org); GeneCards database (https://www.genecards.org/), OMIM database (https://www.omim.org/), PharmGkb database (https://www.pharmgkb.org/); functional protein contact network (STRING) database (https://string-db.org/); Cytoscape 3.7.1 (https://cytoscape.org/); R language software Rx64 3.6.2 (https://www.r-project.org/); Bioconductor (https://www.bioconductor.org/), an R plug in source; and Microsoft Windows 10 Home operating environment.

### 2.2. Collection of Bioactive Compounds of Coptis chinensis

TCMSP, a traditional Chinese medicine (TCM) ingredients database, is commonly used for researching Chinese medicine network pharmacology. It contains more than 500 herbal medicines and more than 3,000 kinds of Chinese herbal medicines. It has collected more than 3300 targets of ingredients from databases such as DrugBank and HIT. TCMSP was used to search all the active ingredients in the traditional Chinese medicine, *C. chinensis*. According to the research results, screening was performed with oral bioavailability (OB) ≥ 30% and drug-likeness (DL) ≥ 0.18 as the limiting conditions to obtain the biologically active components of *C. chinensis*.

### 2.3. Prediction of Target of Coptis chinensis

Targets of effective active ingredients of *C. chinensis* were collected from the TCMSP database. For the convenience and standardization of data processing, the Uniprot database was used to uniformly convert the “protein name” of the target into the “gene name.”

### 2.4. Disease Targets for Polycystic Ovary Syndrome

Using the search term “Polycystic Ovary Syndrome,” the databases GeneCards, OMIM, and PharmGkb were interrogated. These platforms are comprehensive disease-related data platforms, and there are a wide range of data related to complex diseases, including data literature, and experimental verifications. Search results from the three platforms were refined to obtain the disease targets of PCOS.

### 2.5. Screening of Common Targets for Drugs and Diseases and Construction of Interaction Networks

R software was used to obtain the common target of drugs and diseases, and a Venn diagram was drawn. The STRING platform was used to construct the common target protein-protein interaction (PPI). The minimum interaction threshold was set to “highest confidence” (>0.9). “Node” was used to represent different targets, and “edge” represented the relationship between different targets. R software was used to count the frequency of occurrence and to produce a histogram. All R packages were downloaded via the R and Bioconductor websites.

### 2.6. Construction of Drug-Active Ingredient-Disease-Target Interaction Network

Cytoscape 3.7.1 (https://cytoscape.org/) software was used to build a drug-active ingredient-disease-target interaction network. In the resulting network diagram, the software defines nodes and edges. “Node” represents drugs, active ingredients, diseases, and targets, and “edge” represents the relationship between the above nodes.

### 2.7. Gene Ontology (GO) Biological Function Analysis and Kyoto Encyclopedia of Genes and Genomes (KEGG) Path Enrichment Analysis

GO and KEGG data were analyzed using OmicShare tools (htps://www.omicshare.com/tools), and the results were presented as bubble charts. P was defined as significant at a value < 0.05. The degree of enrichment of core pathways was analyzed based on the value of the enrichment factor to explore the possible mechanism of *C. chinensis* in treating PCOS. In addition, the ClueGo plugin in Cytoscape 3.7.1 software was used to perform GO analysis on common targets of drugs and diseases, and the results were presented as an interaction network and a pie chart.

### 2.8. Molecular Docking

The interaction between active components and their targets was evaluated to explore the binding modes; four components and four targets were chosen. PubChem (https://pubchem.ncbi.nlm.nih.gov/) was used to collect the 3D structure data of quercetin (CID 5280343), berberrubine (CID 72703), berberine (CID 2353), and canadine (CID 34458). PDB data were used for the structures of MAPK1 (PDBID: 6G54), CXCL8 (PDBID: 5D14), IL-6 (PDBID: 4O9H), and IL-1*β* (PDBID: 4G6M). Schrodinger software (v9.2; https://www.schrodinger.com/) was used to pretreat compound structures and generate multiple conformations. Protein structures were processed by removing hydrone and adding hydrogen atoms, and the Sitemap module was used to explore and define binding sites. The ligand-dock module was used to simulate the molecular docking of compounds and proteins, and the docking score was evaluated using the Docking score function. Simultaneously, the visual analysis module was used to observe the binding postures of molecules and their targets.

### 2.9. *Coptis chinensis* Granules (CCG) Preparation

The CCG was composed of *Coptis chinensis* (10g). And the dry whole body of *Coptis chinensis* granules was provided by the department of TCM granules in Zhejiang Integrated Traditional and Western Medicine Hospital according to the Chinese Pharmacopoeia (2015) and prepared in the Huisong Pharmaceuticals Co., Ltd (Zhejiang, China). The final prepared WZYZP was sealed and stored in a dry and cool location.

### 2.10. UHPLC-QTOF-MS/MS Analysis of *C. chinensis*

Ultra-high-performance liquid chromatography quadrupole time-of-flight mass spectrometry (UHPLC-QTOF-MS/MS) was used to verify the *C. chinensis* composition. Chromatographic analysis was performed using an ACQUITY BEH C18 column (100 × 2.1 mm, 1.7 *μ*m) on a Waters ACQUITY UHPLC system with a binary solvent manager at a flow rate of 0.3 mL/min, sample injection volume of 1.0 *μ*L, and column temperature of 30°C. The MS analysis was conducted on a Waters G2 QTOF TM system (Waters MS Technologies, Manchester, UK) operated using software.

### 2.11. Experiments and Ethnic Statements

Healthy female C57BL/6N mice (specific-pathogen free, SPF), weighing 16–20 g, were obtained from the Animal Center of Zhejiang Chinese Medical University (Hangzhou, Zhejiang, China), certificate No.: SYXK (Zhejiang province) 2021–0012. The experimental procedures were performed based on the Zhejiang Chinese Medical University Committee on Laboratory Animals (protocol number 202103-0079 and 20210308-05). All mice were maintained under the conditions (at 22 ± 2°C, relative humidity 50 ± 10%, 12-hour light/dark cycle) and were provided water and chow ad libitum in the laboratory environment.

### 2.12. Study Procedure

A total of 24 female C57BL/6N mice were randomly labeled into 4 groups as follows: normal control group, PCOS group, PCOS + *Coptis chinensis* 2.7 group (*C. chinensis* 2.7 group) and PCOS + *Coptis chinensis* 5.4 group (*C. chinensis* 5.4 group), 6 mice each group. The PCOS and PCOS + *Coptis chinensis* (2.7 and 5.4 g/kg groups) were established by the oral administration of letrozole solution at the dose of 1.0 mg/kg for 25 days. In addition, the normal control group was orally given normal saline. At the beginning of the experiment, the mice were weighed every other day, and the changes in body weight were observed for 21 days. After the establishment of the PCOS model, mice in the PCOS + *Coptis chinensis* (2.7 and 5.4 g/kg groups) were treated with *Coptis chinensis* 2.7 g/Kg/d and 5.4 g/Kg/d for 25 days.

### 2.13. Morphological Observation

Using hematoxylin and eosin (H&E) staining, ovarian morphology was observed. Ovarian tissues were fixed in 4% paraformalin overnight at room temperature, embedded in paraffin, and cut into several 4 *μ*m sections. Next, the slides were stained by hematoxylin and eosin. Subsequently, the slices were dehydrated in 95, 90, and 70% ethanol, cleared in xylene. Finally, the slides were observed under the microscope (Olympus DP73, Tokyo, Japan).

### 2.14. Quantitative Reverse Transcription Polymerase Chain Reaction (qRT-PCR)

Use the reverse transcription method to prepare cDNA (needed for experiment) according to the manufacturer's instructions. The expected detection indicators in this experiment are MAPK1, CXCL8, IL-6, and IL-1*β*. The forward and reverse sequences are described in Supplementary Table 1 (Supporting Information). The reaction was carried out on a fluorescent quantitative PCR machine, and all samples were repeated in three parallel wells. At the end of the experiment, record the number of cycles (Ct value), in which the fluorescence signal in each reaction tube reaches the set threshold. In order to compare the difference in gene expression, relative quantification (RQ method [RQ = 2 ΔΔCt]) is used to calculate the multiple of the target gene relative to the reference gene.

### 2.15. Western Blot

100 mg of ovarian tissue from each of the four groups was homogenized with RIPA lysis buffer for 30 minutes on ice, and the total protein concentration was measured using the bicinchoninic acid assay. Separate the protein sample (50 *μ*g) using SDS-PAGE and electrotransfer to a polyvinylidene fluoride membrane. After blocking with 5% skim milk, the membrane was incubated overnight at 4°C with the following antibodies: anti-MAPK1 (CST, USA), anti-CXCL8 (Affinity, USA), anti-IL-6 (Affinity, USA), anti-IL-1*β* (Affinity, United States), and anti-GADPH (Affinity, United States). The membrane was further incubated with the secondary antibody; ECL-Plus chemiluminescence was used to detect immunoreactive bands.

### 2.16. Statistical Analysis

The experimental data are presented as the mean ± standard deviation (SD). Statistical analysis was performed with SPSS 18.0 software. Statistical significance between groups was calculated by one-way analysis of variance (ANOVA) followed with post hoc LSD test. *P* < 0.05 was considered to be statistically significant.

## 3. Results

### 3.1. Screening of Active Components of Coptis chinensis

The TCMSP database contained 48 species of *C. chinensis*. According to the screening conditions, there were 26 components with OB ≥ 30%, 31 kinds with DL ≥ 0.18, and 14 kinds of effective serum active ingredients with OB ≥ 30% and DL ≥ 0.18. These included berberine, obacunone, berberrubine, epiberberine, canadine, berlambine, corchoroside A, magnograndiolide, palmidin A, palmatine, quercetin, coptisine, worenine, and moupinamide (Table 1).

### 3.2. Collection of Targets for Effective Active Ingredients of *Coptis chinensis*

The component target model in the TCMSP database was searched, and there were 581 component targets for *C. chinensis*. Among them, hydrogenated berberine acted on 31 targets, berberine acted on 16 targets, berberine acted on 13 targets, and quercetin acted on 139 targets, which are the more effective targets. The Uniprot database was used to collect the gene names of the active targets and delete invalid and duplicate targets, leading to a total of 148 active targets of C. chinensis.

### 3.3. Collection of Polycystic Ovary Syndrome-Related Genes

**Figure 2:**
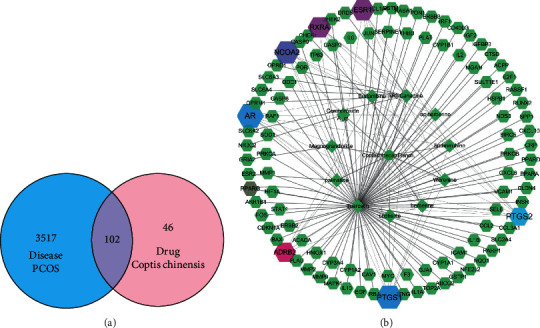
The Venn diagram of overlapping genes and Network of Drug-Ingredients-Genetics. (a) The 102 overlapping genes between the drug and disease. (b) Network of Drug-Ingredients-Genes. The 102 hexagonal nodes represent the overlapping genes between the drug and disease. The 14 diamond-shaped nodes represent the active ingredients in *C. chinensis*. The edges in the figure denote the nodes that can interact with each other.

The Genecards, OMIM, and PharmGkb disease databases were searched, and a total of 3,517 disease targets related to PCOS were collected. The R language was used to map the active targets of the active ingredients of *C. chinensis* to these disease targets. A Venn diagram showed that a total of 102 intersecting targets were obtained, which were defined as the “key targets” for *C. chinensis* to treat PCOS (Figure 2). Among them, mitogen-activated protein kinase (MAPK1), insulin-like growth factor 2 (IGF2), and protein kinase B1 (Akt1), *inter alia*, were of significance in the occurrence and development of PCOS.

### 3.4. Construction of a Common Target Interaction Network

**Figure 3:**
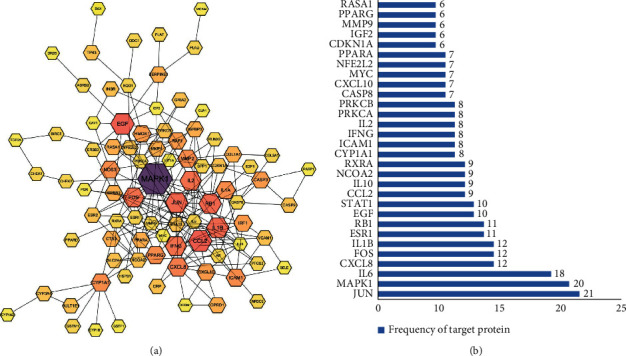
The protein-protein interaction (PPI) network. (a) Target protein-protein interaction network (PPI) of biologically active ingredients related to the treatment of polycystic ovary syndrome in *C. chinensis*. Each node represents a related target gene. A greater degree of target PPI is represented by darker colors and larger nodes, and edges with higher merge scores are denoted by darker and thicker lines. (b) Bar graph of the frequency of PPI network target proteins. The *x*-axis represents the number of target proteins connected to neighboring proteins, whereas the *y*-axis represents the top 30 target proteins with higher interaction frequencies.

**Figure 4:**
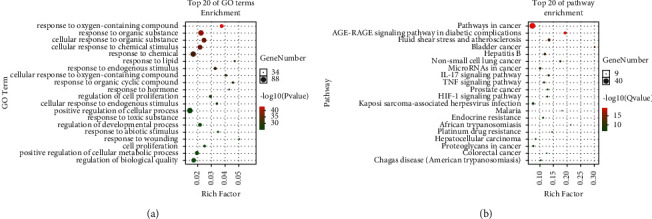
Enrichment analysis of gene ontology (GO) biological processes and KEGG signaling pathway enrichment analyses. (a) GO tool-based analysis of 102 genes related to polycystic ovary syndrome. The *x*-axis represents the enrichment factor of the target gene, and the *y*-axis represents the “biological process” category, where the target gene is enriched in GO (*P* < 0.01). The size of the bubble area represents the number of genes belonging to the GO item in the target gene. The color of the bubble indicates the concentration, and a darker color indicates a higher importance. (b) KEGG signal pathway enrichment analysis. The *x*-axis represents the enrichment factor of the target gene, whereas the *y*-axis represents the main signal pathway (*P* < 0.01); the size of the bubble in the figure represents the number of genes belonging to the pathway in the target gene. The color of the bubble indicates the importance of enrichment; a red color indicates a higher importance.

There were 102 nodes and 221 edges in the interaction network (Figure 3). The frequency of occurrence of the 30 targets is shown in Figure 4. Frequent protein interactions included v-jun avian sarcoma virus 17 oncogene homolog (JUN), mitogen-activated protein kinase (MAPK), interleukin (IL-6), recombinant human interleukin 8 (CXCL8), FOS, and a recombinant human interleukin 1 B (IL1B). Nodal proteins (indicating that the active components of *C. chinensis* had a high binding activity with them) can be used as potential targets for *C. chinensis* in treating PCOS.

### 3.5. Construction of a Drug-Active, Ingredient-Disease-Target, Interaction Network

**Figure 5:**
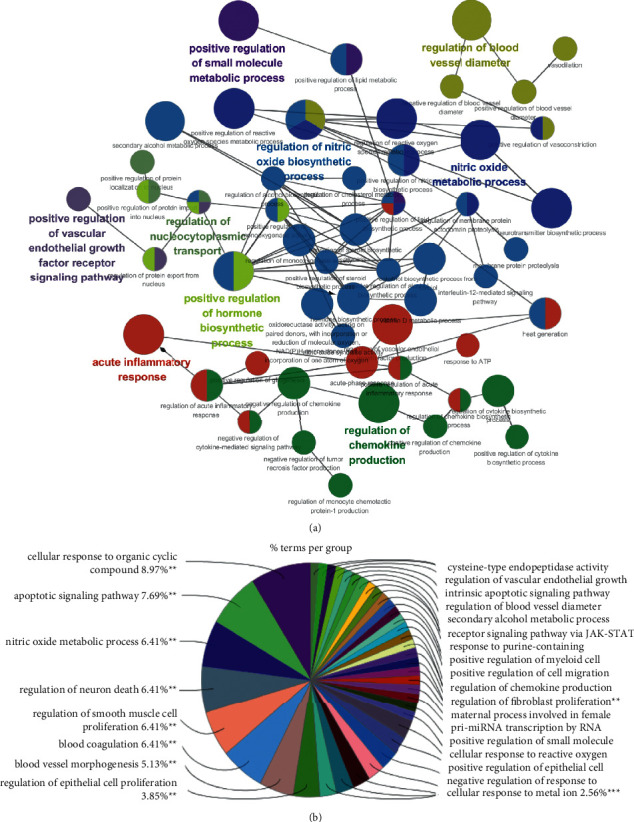
Biological function classification of 102 genes related to polycystic ovary syndrome using the Cluego tool. (a) The Cluego tool analyzed 102 genes related to polycystic ovary syndrome. Different node colors represent the biological process of enrichment. The size of the node indicates the degree of enrichment. The larger the node, the higher the degree of enrichment. The connections are represented by a line. (b) Enrichment analysis of the gene ontology (GO) terms. The area of the pie chart represents the proportion of the target genes enriched in different biological processes.

The components of *C. chinensis*, the target on which the component act, and the target of the disease were input into Cytoscape 3.7.1 to build a drug-active, component-disease-target, interaction network (Figure 5). The network diagram contained 115 nodes, of which 102 nodes represented the target of the action; there were 259 edges indicating the relationship between nodes. The network showed that PTGS2, PTGS1, AR, and NCOA2 were linked to 9, 8, 8, and 7 components, respectively, and may serve as the central genes for *C. chinensis* to treat PCOS. Quercetin was linked to 91 genes, indicating that it may be the key active ingredient.

### 3.6. Screening of Core Pathways for *Coptis chinensis* in Treating Polycystic Ovary Syndrome

To determine whether the 102 identified genes were related to PCOS, a GO pathway enrichment analysis was performed to elucidate the relevant biological processes (*P* < 0.01). A bubble chart was drawn, using the OmicShare tools (http://www.omicshare.com/tools) (Figure 4(a)). The GO analysis indicated that biological processes such as apoptosis, cytokine activation, oxidant antioxidant, and nitric oxide metabolism were mainly involved. *C. chinensis* may, therefore, treat PCOS by regulating multiple complex biological processes. Similarly, the KEGG pathway enrichment analysis of the above common targets was performed with the help of this network platform (Figure 4(b)). Excluding extensive pathways, the top 20 signaling pathways are listed in Table 2. The 102 common targets were mainly distributed in multiple signaling pathways such as AGEs/RAGE, MAPK, PI3k/Akt, and TNF, which suggests that *C. chinensis* treats PCOS by acting on multiple pathways and that there is a gap between these pathways, indicating complex interactions. The size of the node indicates the number of enriched targets, and the color of the node from green to red indicates that the *P* value changes from large to small. Therefore, the larger the red node, the higher the significance of the signal path.

In the Cytoscape 3.7.1 (https://cytoscape.org/) software, the ClueGo plugin was used to perform GO term analysis on the common targets, and an interactive network and pie chart were obtained. The degree of correlation between the enriched biological processes and the number of genes included was described previously (Figures 5(a) and 5(b)).

### 3.7. Molecular Docking Results

**Figure 6:**
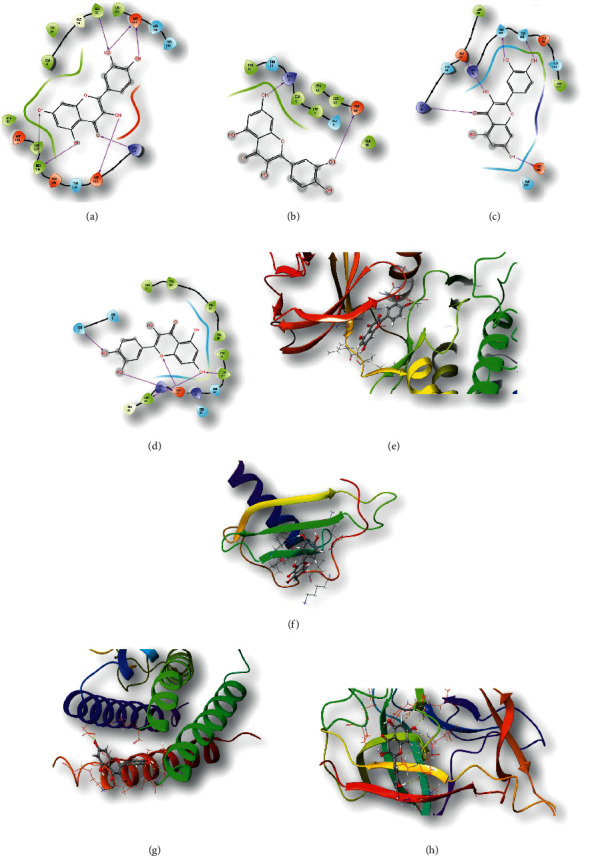
Molecular docking between quercetin and related targets. (a, e) Two-dimensional and three-dimensional results of virtual molecular docking of MAPK1 and quercetin, respectively. (b, f) Two-dimensional and three-dimensional results of virtual molecular docking of CLCX8 and quercetin, respectively. (c, g) Two-dimensional and three-dimensional results of virtual molecular docking of IL-6 and quercetin, respectively. (d, h) Two-dimensional and three-dimensional results of virtual molecular docking of IL-1*β* and quercetin, respectively.

Molecular docking simplifies the study of the possible interaction modes of compounds and hub genes. The number of ligands bound to the receptor and its binding strength depend on the inhibition efficiency. Therefore, the interaction and binding mode of active ingredients (quercetin, berberine, berberrubine, and canadine) and cellular inflammatory pathway related factors (MAPK1, CXCL8, IL-6, and IL-1*β*) were studied through molecular docking. A score of <5 points indicates that the compound has excellent docking activity with the target. Docking scores of quercetin with MAPK1 (−6.894), CXCL8 (−5.185), IL-6 (−5.864), and IL-1*β* (−6.048) were the highest among the selected components. Quercetin forms covalent bonds with the ALA35, ASP111, ASP167, MET108, and LYS114 amino acid residues of MAPK1 (Figures 6(a) and 6(e)) and forms covalent bonds with the LYS9 and GLU16 amino acid residues of CXCL8 (Figures 6(b) and 6(f)). It forms covalent bonds with the LYS46, GLU42, SER107, SER108, and ASP160 amino acid residues of IL-6 (Figures 6(c) and 6(g)) and forms covalent bonds with the LEU62, LEU67, and GLU64 amino acid residues of IL-1*β* (Figures 6(d) and 6(h)), indicating that the combination is relatively stable. The molecular docking results between the remaining active ingredients and the target gene are shown in Table 3 and Figure S1.

### 3.8. Chemical Characterization of Coptis chinensis

**Figure 7:**
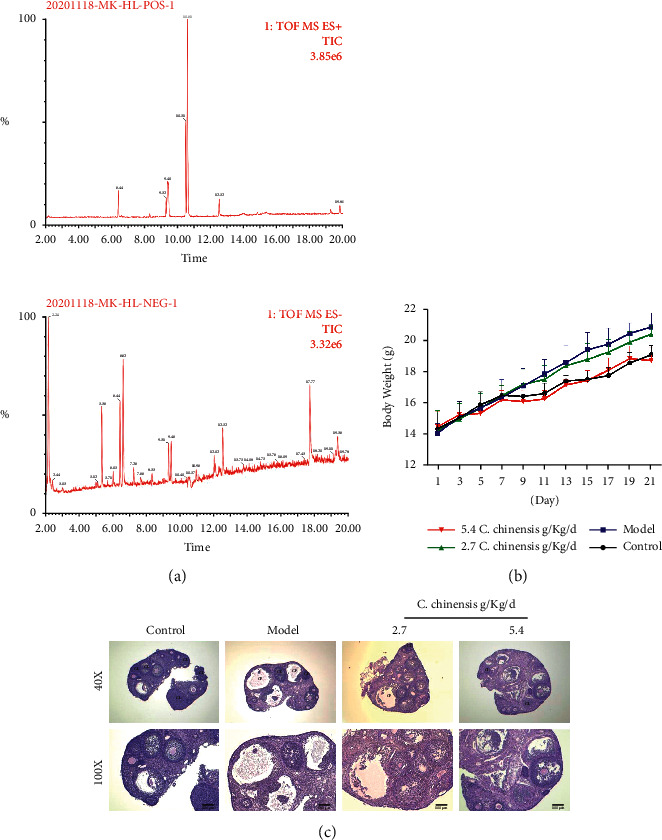
Compounds identification and the effect of the *C. chinensis* on the experimental PCOS model mice. (a) The phytochemical compositions identification in the WZYZP by UHPLC-QTOF-MS/MS in the positive ion mode and negative ion mode. (b) Effect of the different doses of *C. chinensis* on body weight of mice. (c) HE staining to evaluate the effect of the *C. chinensis* on mouse ovarian histological changes, above magnification ×40, 100.

The chemical characterization of Coptis chinensis was analyzed with UHPLC-QTOF-MS/MS, and the results are presented in Figure 7(a). The obtained mass data was analyzed using SCIEX OS software. According to the first-order accurate mass number, isotope distribution ratio, and MS/MS data of the compounds and the TCM MS/MS library in SCIEX OS software, 8 compounds were identified under positive ion mode, and 2 compounds were identified under the negative ion mode from *Coptis chinensis*. Some of compounds were identified under both positive ion mode and negative ion mode. These compounds are mainly alkaloids, and a small part are acids. In addition, some of the compounds identified by UHPLC-QTOF-MS/MS were coincident with the aforementioned compounds screened from the TCMSP database in Coptis chinensis.

### 3.9. The Effect of CCG on Body Weight and Ovarian Tissue of PCOS Mice

We found that, compared with the weight of the normal group of mice, the model group had a higher weight than the normal group from day 9, and the difference was statistically significant (*P* < 0.05). However, there was no significant difference in body weight between the model group and the treatment group (Figure 7(b)). In addition, we observed that, compared with normal mice, the PCOS group mice had more cystic dilated follicles (CF), significantly reduced granulosa cell layer, and decreased corpus luteum (CL). Compared with the PCOS group, the number of cystic dilated follicles (CF) in the two doses of *Coptis* treatment group decreased, and the number of granulosa cell layers and the corpus luteum (CL) increased (Figure 7(c)).

### 3.10. The Effect of CCG on the Concentration of MAPK1, CXCL8, IL-6, and IL-1*β* in the Ovarian Tissue of PCOS Mice

**Figure 8:**
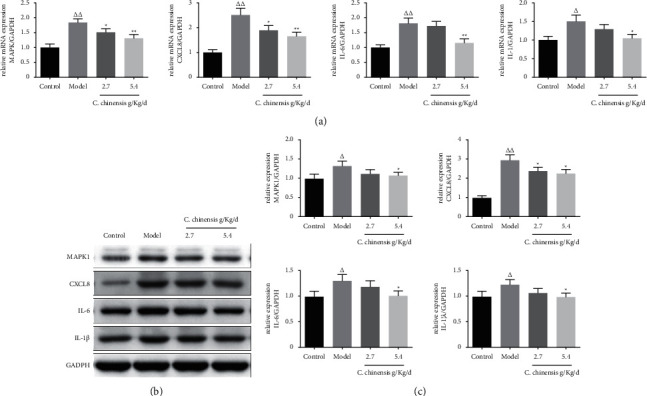
Effect of the *C. chinensis* on the hub targets and inflammation pathway. (a) mRNA expression of MAPK1, CXCL8, IL-6, and IL-1*β* in mouse ovarian. (b) Protein expression of MAPK1, CXCL8, IL-6, and IL-1*β* in mouse ovarian. (c) Statistical graph of protein expression of MAPK1, CXCL8, IL-6, and IL-1*β*. The protein expression was detected with a western blot assay. GADPH was used as control. ^Δ^*P* < 0.05 vs Control group, ^ΔΔ^*P* < 0.01 vs Model group, ^*∗*^*P* < 0.05 vs Control group,  ^*∗∗*^*P* < 0.01 vs Model group.

Combining the PPI network interaction targets and molecular docking results of the common drug-disease target, we selected inflammation-related genes as verification targets and used qRT-PCR to detect the expression levels of MAPK1, CXCL8, IL-6, and IL-1*β* mRNA (Figure 8(a)). We observed that, after modeling, the expression of MAPK1, CXCL8, and IL-6 mRNA in the PCOS group was significantly higher than that in the NC group (*P* < 0.01), and the mRNA expression of IL-1*β* was also significantly higher than that in the NC group (*P* < 0.05). After drug intervention, the mRNA expression of MAPK1 and CXCL8 in the PCOS + *C. chinensis* 2.7 g group was lower than that in the PCOS group (*P* < 0.05); the mRNA expression of MAPK1, CXCL8, and IL-6 in the PCOS + *C. chinensis* 5.4 g group was significantly lower than that in the PCOS group (*P* < 0.01), and the mRNA expression of IL-1*β* was also lower than that of the PCOS group (*P* < 0.05). In addition, we used Western Blot to detect MAPK1, CXCL8, IL-6, and IL-1*β* in mouse ovarian tissue Protein expression level (Figures 8(b) and 8(c)). We observed that, after modeling, the protein expressions of MAPK1, CXCL8, IL-6, and IL-1*β* in the model group were significantly higher than those in the NC group (*P* < 0.05, *P* < 0.01, *P* < 0.05, *P* < 0.05). After treatment, compared with the PCOS model group, the relative protein expression levels of CXCL8 in the PCOS + *C. chinensis* 2.7 g group were significantly reduced (*P* < 0.05), and the relative protein expression levels of MAPK1, CXCL8, IL-6, and IL-1*β* in the PCOS + *C. chinensis* 5.4 g group were significantly reduced (*P* < 0.05).

## 4. Discussion

Polycystic ovary syndrome is an endocrine syndrome associated with ovulatory disorders and metabolic abnormalities. Since the main external characteristics of the disease are infrequent menstruation and prolonged infertility, in TCM, PCOS is mostly classified as “late menstruation,” “less menstruation,” “amenorrhea,” “collapse,” and “infertility.” The etiology and pathogenesis of the disease (in TCM) involve kidney-Tiangui-Chongren-cycle axis dysfunction, phlegm dampness, and obstruction in the middle focus, with heat over time, resulting in kidney, liver, and spleen dysfunction. *Coptis chinensis* is a representative medicine for clearing away heat and dampness in TCM. At present, most clinical studies focus on the effect of the berberine extract component (“berberine”), but pharmacological studies have found that a Huang Lian (Chinese Herb) decoction is better than berberine monomers in improving insulin resistance, lowering blood sugar, and improving glucose and lipid metabolism disorders [12, 13]. Due to the complex composition of TCM *C. chinensis*, many ingredients often participate in the process of exerting its effects, and the mechanisms of action are intertwined with each other. Therefore, it is necessary to conduct a comprehensive analysis on the relevant network pharmacology software platforms to interpret the mechanisms of action.

In this study, the main active ingredients of *C. chinensis* were found to include various organic components such as berberine, berberine, quercetin, canadine, and berberrubine. The core nodes in the “target” network suggest that these several active ingredients play an extremely important role in the treatment of PCOS. Other related studies have also verified that several components in *C. chinensis* improve follicular developmental disorders, ovulatory disorders, and lower fasting blood glucose and fasting insulin levels to reduce insulin resistance in women with PCOS [14, 15], concurring with the current study. A large, multicenter, randomized, double-blind, placebo-controlled trial of berberine in PCOS showed that berberine combined with the first-line, ovulation-promoting agent, letrozole, can improve ovulation rate and pregnancy [16]. In addition, berberine has the advantage of lowering the incidence of adverse gastrointestinal reactions and severe ovarian hyperstimulation syndrome, compared with letrozole.

Through analysis of drug and disease targets, it was found that quercetin has 139 targets, which are active ingredients with many targets. Quercetin is a natural flavonoid, which can inhibit the activity of aldose reductase and has the effect of reducing the advanced glycation end products (AGEs) and insulin resistance. It has attracted the attention of researchers and clinicians [17]. The use of quercetin in the treatment of type 2 diabetes is becoming increasingly common. Treatment of diabetic rats with quercetin and metformin improved insulin sensitivity and increased liver glycogen levels, suggesting that the two substances act in a similar way [14]. In addition, quercetin can change the body composition of patients with obesity and dyslipidemia, thereby improving body metabolism and hormone levels [7]. Therefore, quercetin is also gradually applied to improve the treatment of patients with PCOS.

Analysis of the PPI network indicated that MAPK, JUN, IL-6, CXCL8, and FOS are at the core of the network and can be regarded as potential key targets for *C. chinensis* in treating PCOS. A mitogen-activated protein kinase pathway (MAPK) is involved in the regulation of oocyte maturation, and this effect is essential for normal follicular development and proper ovulation [18]. Once the MAPK pathway is blocked, mitotic cyclin D2 expression and the role of granulocyte proliferation will be impeded, resulting in a decrease in the number of granulocytes, which indirectly interferes with the development and maturation of follicles [19, 20]. Experiments have confirmed that the high expression of IL-6 in PCOS may play a role in the pathogenesis of PCOS through the synergistic effect of insulin resistance and chronic inflammation [21]. The abnormally low expression of the *FOS* gene in granular cells may increase the androgen secretion of ovarian granular cells by reducing the inhibitory effect on the *CYP17* gene [22]. The results of molecular docking showed that the active ingredient, quercetin, with the most targets, had a higher degree of binding to MAPK, IL-6, CXCL8, and IL-1*β*, suggesting that the compound may play a therapeutic role by combining with these proteins.

Further experimental studies on C57BL/6N model mice show that *C. chinensis* can alleviate ovulation disorders in mice, improve the quality of follicles, and reverse ovarian histopathological damage. *C. chinensis* also significantly reduces the expression of inflammatory factor proteins and mRNA and reduces the level of cellular inflammation in ovarian tissue. These results indicate that *C. chinensis* has a therapeutic effect on polycystic ovary syndrome by reducing ovarian inflammation. And we speculate that the biologically active compounds screened from *C. chinensis* may significantly promote this effect, which requires further verification by subsequent experiments.

ClueGO biological process enrichment analysis was performed on the key targets of the active components of *C. chinensis*, which confirmed that *C. chinensis* has certain effects on biological processes such as apoptosis, cytokine activation, oxidant antioxidant, and nitric oxide metabolism. The process is reflected in the targets and related pathways involved in the treatment of PCOS by *C. chinensis*. In the KEGG enrichment analysis, *C. chinensis* was shown to regulate various PCOS pathogenesis-related signaling pathways including AGEs/RAGE, MAPK, PI3k/Akt, and TNF. Studies have shown that the high expression of AGEs plays a key role in the formation of PCOS. AGEs mainly bind to specific receptors (RAGE) and mediate a series of pathological responses through cellular signal transduction mechanisms have found that, after binding to AGEs, RAGE can inhibit the activation of phosphatidylinositol 3-kinase (PI3K/Akt) and the expression of related genes in the course granule cells, so that the glucose transporter (GLUT4) can be transferred to the plasma membrane. Obstacles, inhibition of glycogen synthesis, inhibition of glycolysis, promotion of gluconeogenesis, inhibition of protein synthesis, and changes in gene expression promote elevated blood glucose, severe hyperinsulinemia, and IR. In the process of follicular development, oocytes find it difficult to synthesize glucose, and most of their energy comes from granulocyte glycolysis [24]. Therefore, AGEs/RAGE may reduce glucose intake by reducing the number of granulocyte membrane GLUT⁃4 receptors and thereby reduce glucose intake, affecting oocyte growth [20]. In addition, upregulated RAGE may reduce the sensitivity of granulocytes to follicle stimulating hormone (FSH) by inhibiting the MAPK/ERK 1/2 pathway and promoting increased apoptosis of granulosa cells [18, 19]. The results of this study reflect the characteristics of the multicomponent, multitarget, and multipath treatment of diseases with botanicals and provide a reasonable reference for clarifying the mechanism of the botanical *Coptis chinensis* in treating PCOS.

## 5. Conclusions

Our research uses a combination of methods including network pharmacology, molecular docking, and in vivo experiments to systematically explore the mechanism of action of *C. chinensis* in the treatment of PCOS. Through bioinformatics analysis, a total of 102 common targets related to *C. chinensis* and PCOS were identified. Network analysis identified MAPK1, JUN, IL-6, CXCL8/IL-8, FOS, IL1B, ESR1, and EGF as the central targets and revealed that the cellular inflammation pathway is a key pathway, which has multiple signaling pathways and multiple targets. The molecular docking results showed that the biologically active components in *C. chinensis* inhibit cell inflammation by regulating the expression of MAPK1, IL-6, CXCL8, and IL1B, thereby exerting a therapeutic effect on PCOS. In addition, in vivo experiments have shown that *C. chinensis* treatment can improve polycystic ovary syndrome by improving the quality of follicles and reversing ovarian histopathological damage, reducing the expression of cellular inflammation-related proteins (MAPK1, IL-6, CXCL8, and IL1B) in ovarian tissue. Therapeutic effect: based on network pharmacological analysis and experimental verification, this study shows that *C. chinensis* is effective in reversing polycystic ovary syndrome, suggesting that it may be an alternative to the treatment of PCOS. However, further in vitro and in vivo studies are still needed to clarify the role and complex mechanisms of *C. chinensis* in the treatment of polycystic ovary syndrome.

## Figures and Tables

**Table 1:** Active ingredients of *Coptis chinensis* selected from TCMSP.

Molecular number	Active ingredients	Chemical formula	OB %	DL
MOL001454	Berberine	C^20^H^18^ClNO^4^	36.86	0.19
MOL013352	Obacunone	C^26^H^30^O^7^	43.29	0.31
MOL002894	Berberrubine	C^19^H^16^ClNO^4^	35.74	0.24
MOL002897	Epiberberine	C^20^H^17^NO^5^	43.09	0.19
MOL002903	(R)-Canadine	C^20^H^21^NO^4^	55.37	0.2
MOL002904	Berlambine	C^20^H^17^NO^5^	36.68	0.28
MOL002907	Corchoroside A	C^29^H^42^O^9^	104.95	0.29
MOL000622	Magnograndiolide	C^15^H^22^O^4^	63.71	0.3
MOL000762	Palmidin A	C^30^H^22^O^8^	35.36	0.39
MOL000785	Palmatine	C^21^H^24^ClNO^5^	64.6	0.13
MOL000098	Quercetin	C^15^H^10^O^7^	46.43	0.38
MOL001458	Coptisine	C^19^H^14^ClNO^4^	30.67	0.26
MOL002668	Worenine	C^20^H^16^NO^4+^	45.83	0.27
MOL008647	Moupinamide	C^18^H^19^NO^4^	86.71	0.33

The table lists the effective ingredients of *Coptis chinensis* obtained in the TCMSP database with OB ≥ 30 and DL ≥ 0.18 as the screening conditions. TCMSP: TCM systems pharmacology database and analysis platform. OB: Oral bioavailability. DL: Drug-like.

**Table 2:** Result of target pathway enrichment (top 20).

Pathway ID	Pathway name	Gene Name	Count	*P* value
hsa04933	AGE-RAGE signaling pathway in diabetic complications	*BAX/MMP2/MAPK1/JUN/IL6/CASP3/PRKCA/STAT1/F3/ICAM1/IL1B/CCL2/SELE/VCAM1/CXCL8/PRKCB/NOS3/THBD/SERPINE1/COL1A1/IL1A/COL3A1*	22	3.46*E* − 22
hsa04010	MAPK signaling pathway	*FOS/MAPK1/EGF/JUN/CASP3/RAF1/PRKCA/ERBB2/MYC/IL1B/PRKCB/HSPB1/IL1A/INSR/IGF2/ERBB3/RASA1*	17	1.04*E* − 07
hsa04151	PI3K-Akt signaling pathway	*RXRA/CDKN1A/CASP9/MAPK1/EGF/IL6/RAF1/PRKCA/ERBB2/MYC/NOS3/IL2/COL1A1/INSR/SPP1/IGF2/ERBB3*	17	1.38*E* − 06
hsa04668	TNF signaling pathway	*PTGS2/FOS/MMP9/MAPK1/JUN/IL6/CASP3/CASP8/ICAM1/IL1B/CCL2/SELE/VCAM1/CXCL10/IRF1*	15	5.17*E* − 12
hsa04657	IL-17 signaling pathway	*PTGS2/FOS/MMP9/MAPK1/JUN/IL6/CASP3/CASP8/MMP1/IL1B/CCL2/CXCL8/IFNG/CXCL10*	14	5.44*E* − 12
hsa01522	Endocrine resistance	*ESR1/ESR2/FOS/CDKN1A/BAX/MMP2/MMP9/MAPK1/RB1/JUN/RAF1/ERBB2/E2F1*	13	1.71*E* − 10
hsa04066	HIF-1 signaling pathway	*CDKN1A/MAPK1/EGF/IL6/PRKCA/HIF1A/ERBB2/HMOX1/PRKCB/NOS3/SERPINE1/IFNG/INSR*	13	6.65*E* − 10
hsa04218	Cellular senescence	*CDKN1A/MAPK1/RB1/IL6/RAF1/MYC/CXCL8/SERPINE1/IL1A/CHEK2/E2F1/IGFBP3/CHEK1*	13	7.42*E* − 08
hsa04915	Estrogen signaling pathway	*ESR1/NCOA2/OPRM1/ESR2/FOS/MMP2/MMP9/MAPK1/JUN/RAF1/NOS3/CTSD*	12	1.16*E* − 07
hsa04625	C-type lectin receptor signaling pathway	*PTGS2/MAPK1/IL10/JUN/IL6/CASP8/RAF1/STAT1/IL1B/IL2/IRF1*	11	5.36*E* − 08
hsa04919	Thyroid hormone signaling pathway	*ESR1/RXRA/NCOA2/CASP9/MAPK1/RAF1/PRKCA/HIF1A/STAT1/MYC/PRKCB*	11	2.17*E* − 07
hsa04926	Relaxin signaling pathway	*FOS/MMP2/MMP9/MAPK1/JUN/RAF1/PRKCA/MMP1/NOS3/COL1A1/COL3A1*	11	5.35*E* − 07
hsa04210	Apoptosis	*FOS/BAX/CASP9/MAPK1/JUN/CASP3/CASP8/RAF1/BIRC5/PARP1/CTSD*	11	8.43*E* − 07
hsa04012	ErbB signaling pathway	*CDKN1A/MAPK1/EGF/JUN/RAF1/PRKCA/ERBB2/MYC/PRKCB/ERBB3*	10	8.06*E* − 08
hsa04620	Toll-like receptor signaling pathway	*FOS/MAPK1/JUN/IL6/CASP8/STAT1/IL1B/CXCL8/CXCL10/SPP1*	10	5.54*E* − 07
hsa04659	Th17 cell differentiation	*RXRA/FOS/MAPK1/JUN/IL6/HIF1A/STAT1/IL1B/IL2/IFNG*	10	7.23*E* − 07
hsa04115	p53 signaling pathway	*CDKN1A/BAX/CASP9/CASP3/CASP8/SERPINE1/CHEK2/IGFBP3/CHEK1*	9	2.19*E* − 07
hsa04064	NF-kappa B signaling pathway	*PTGS2/PLAU/ICAM1/IL1B/VCAM1/CXCL8/PRKCB/PARP1/CD40LG*	9	3.68*E* − 06
hsa04921	Oxytocin signaling pathway	*PTGS2/FOS/CDKN1A/MAPK1/JUN/RAF1/PRKCA/PRKCB/NOS3*	9	0.000112
hsa04630	JAK-STAT signaling pathway	*CDKN1A/IL10/EGF/IL6/RAF1/STAT1/MYC/IL2/IFNG*	9	0.000173

**Table 3:** Molecular docking score between active ingredients of *C. chinensis* and related targets.

Active ingredients	MAPK1 (6G54)	CXCL8 (5D14)	IL-6 (4O9H)	IL-1*β* (4G6M)
Quercetin (CID 5280343)	−6.894	−5.185	−5.864	−6.048
Berberine (CID 2353)	−4.812	−3.442	−3.084	−3.953
Canadine (CID 34458)	−4.316	−3.581	−4.900	−4.261
Berberrubine (CID 72703)	−5.694	−3.431	−3.605	−5.357

## Data Availability

All the data generated or analyzed during this study are included in this published article and its additional files.

## Abbreviations

PCOS: Polycystic ovary syndrome
*C. chinensis*: *Coptis chinensis*
TCM: Traditional Chinese medicine
TCMSP: TCM systems pharmacology
PPI: Protein-protein interaction
KEGG: Kyoto Encyclopedia of Genes and Genomes
MAPK1: Mitogen-activated protein kinase.
IGF2: Insulin-like growth factor 2
Akt1: Protein kinase B1
IL-6: Interleukin
CXCL8: Recombinant human interleukin 8
IL1B: Recombinant human interleukin 1B
AGEs: Advanced glycation end products
FSH: Follicle stimulating hormone.

## Supplementary Materials

Supplementary Materials Figures S1. Molecular docking between the active ingredients of *C. chinensis* and related targets. Four bioactive ingredients from *C. chinensis* docking were selected with four targets of PCOS. A, B, C, and D represent the virtual molecular docking results of MAPK1, CLCX8, IL-6, IL-1*β*, and quercetin, respectively. E, F, G, and H represent the virtual molecular docking results of MAPK1, CLCX8, IL-6, IL-1*β*, and berberine, respectively. I, J, K, and L represent the virtual molecular docking results of MAPK1, CLCX8, IL-6, IL-1*β*, and canadine, respectively. M, N, O, and P represent the virtual molecular docking results of MAPK1, CLCX8, IL-6, IL-1*β*, and berberrubine, respectively. In the network diagram, the light green circle represents the drug (*C. chinensis*), the green diamond represents the active ingredient contained in *C. chinensis*, and the green hexagon represents the target, where the active ingredient of the drug is mapped to the target of the disease.

## References

[1] Hadjiconstantinou M., Mani H., Patel N. (2017). Understanding and supporting women with polycystic ovary syndrome: a qualitative study in an ethnically diverse UK sample. *Endocrine Connections*.

[2] Busiah K., Colmenares A., Bidet M. (2017). High prevalence of polycystic ovary syndrome in type 1 diabetes mellitus adolescents: is there a difference depending on the NIH and rotterdam criteria?. *Hormone Research in Paediatrics*.

[3] Ndefo U. A., Eaton A., Green M. R. (2013). Polycystic ovary syndrome: a review of treatment options with a focus on pharmacological approaches. *P & T: A Peer-Reviewed Journal for Formulary Management*.

[4] Hwang K. R., Choi Y. M., Kim J. J. (2013). Effects of insulin-sensitizing agents and insulin resistance in women with polycystic ovary syndrome. *Clinical and Experimental Reproductive Medicine*.

[5] Cai-hong L., Zhou K.-y. (2010). Study advances on the effects and mechanisms of active constituents of Coptidis Rhizoma. *Lishizhen Medicine and Materia Medica Research*.

[6] Pang B., Zhao L.-H., Zhou Q. (2015). Application of berberine on treating type 2 diabetes mellitus. *International Journal of Endocrinology*.

[7] Wei W., Zhao H., Wang A. (2012). A clinical study on the short-term effect of berberine in comparison to metformin on the metabolic characteristics of women with polycystic ovary syndrome. *European Journal of Endocrinology*.

[8] Li L., Li C., Pan P. (2015). A single arm pilot study of effects of berberine on the menstrual pattern, ovulation rate, hormonal and metabolic profiles in anovulatory Chinese women with polycystic ovary syndrome. *PLoS One*.

[9] Geng Q., Guo J., Zhang J., Wang F. (2012). A meta-analysis of the Chinese medicine Bushenhuoxue for treatment of ollgospermia and astheuospermia. *Chinese Journal of Family Planning*.

[10] Dong B., Wang R. H., Liu L. M. (2017). Review on common methods and technologies of quantitative composition-activity relationship research of TCM. *Chinese Journal of Information on Traditional Chinese Medicine*.

[11] Hopkins A. L. (2007). Network pharmacology. *Nature Biotechnology*.

[12] Fu Y., Hu B. R., Tang Q., Fu Q., Zhang Q. Y., Xiang J. Z. (2005). Effect of jatrorrhizine, berberine, Huanglian decoction and compound-mimic prescription on blood glucose in mice. *Chinese Traditional and Herbal Drugs*.

[13] Lai X., Zhang Y., Zheng H. (2010). Study on effective substances of San Huang preparation and its single herbs by serum pharmacochemistry. *World Science and Technology-Modernization of Traditional Chinese Medicine*.

[14] An Y., Sun Z., Zhang Y., Liu B., Guan Y., Lu M. (2014). The use of berberine for women with polycystic ovary syndrome undergoing IVF treatment. *Clinical Endocrinology*.

[15] Li Y., Kuang H., Shen W. (2013). Letrozole, berberine, or their combination for anovulatory infertility in women with polycystic ovary syndrome: study design of a double-blind randomised controlled trial: table 1. *BMJ Open*.

[16] Wu X. K., Wang Y., Liu J. P. (2015). Letrozole, berberine, or a combination for infertility in Chinese women with polycystic ovary syndrome: a multicentre, randomised, double-blind, placebo-controlled trial. *Lancet*.

[17] Rezvan N., Moini A., Janani L. (2017). Effects of quercetin on adiponectin-mediated insulin sensitivity in polycystic ovary syndrome: a randomized placebo-controlled double-blind clinical trial. *Hormone and Metabolic Research*.

[18] Su Y.-Q., Wigglesworth K., Pendola F. L., O’Brien M. J., Eppig J. J. (2002). Mitogen-activated protein kinase activity in cumulus cells is essential for gonadotropin-induced oocyte meiotic resumption and cumulus expansion in the mouse. *Endocrinology*.

[19] Kandaraki E. A., Chatzigeorgiou A., Papageorgiou E. (2018). Advanced glycation end products interfere in luteinizing hormone and follicle stimulating hormone signaling in human granulosa KGN cells. *Experimental Biology and Medicine*.

[20] Kayampilly P. P., Menon K. M. J. (2009). Follicle-stimulating hormone inhibits adenosine 5′-monophosphate-activated protein kinase activation and promotes cell proliferation of primary granulosa cells in culture through an akt-dependent pathway. *Endocrinology*.

[21] Wang J., Zhu L., Hu K. (2017). Effects of metformin treatment on serum levels of C-reactive protein and interleukin-6 in women with polycystic ovary syndrome. *Medicine*.

[22] Sang M., Li J., Zhang Y., Wu X. (2015). The mechanism of c-fos gene regulation of 17-alpha hydroxylase (CYP17) expression and testosterone production in ovarian granulosa cells from polycystic ovary syndrome patients. *Science and Technology Review*.

[23] Stensen M. H., Tanbo T., Storeng R., Fedorcsak P. (2014). Advanced glycation end products and their receptor contribute to ovarian ageing. *Human Reproduction*.

[24] Chang H.-M., Klausen C., Leung P. C. K. (2013). Antimüllerian hormone inhibits follicle-stimulating hormone-induced adenylyl cyclase activation, aromatase expression, and estradiol production in human granulosa-lutein cells. *Fertility and Sterility*.

