# Corrigendum to “Network Pharmacology-Based Strategy for Predicting Active Ingredients and Potential Targets of *Coptis chinensis* Franchin Polycystic Ovary Syndrome”

**DOI:** 10.1155/2021/9798627

**Published:** 2021-11-28

**Authors:** Jian Xiong Ma, Miaoyong Ye, Ke Ma, Kang Zhou, Yingying Zhang, Xiting Wang, Hongxuan Tong

**Affiliations:** ^1^The Second Clinical Medical College, Zhejiang Chinese Medical University, Hangzhou 310053, China; ^2^Department of Andrology, Dongzhimen Hospital, Beijing University of Chinese Medicine, Beijing 100007, China; ^3^Department of Urology, The Affiliated Wenling Hospital of Wenzhou Medical University, Wenling, Zhejiang 317500, China; ^4^School of Traditional Chinese Medicine, Beijing University of Chinese Medicine, Beijing 100029, China; ^5^Institute of Basic Theory for Chinese Medicine, China Academy of Chinese Medical Sciences, Beijing 100700, China

In the article titled “Network Pharmacology-Based Strategy for Predicting Active Ingredients and Potential Targets of *Coptis chinensis* Franchin Polycystic Ovary Syndrome” [1], there are errors in the following sections that were introduced during the preparation of the manuscript.

In the Abstract section, “These results indicated that WZYZP has a protective effect on spermatogenesis disorder, suggesting that it could be an alternative choice for male infertility therapy” should be corrected to “These results indicated that *Coptis chinensis* has a therapeutic effect on polycystic ovary syndrome, suggesting that it could be an alternative choice for polycystic ovary syndrome.”

In Section 2.9, “WZYZP” should be corrected to “*Coptis chinensis*.”

In the legend of Figure 7, “WZYZP” should be corrected to “*Coptis chinensis*.”

The authors apologise for these errors and confirm that this does not affect the results or conclusions of the article.
