# Supplementary material for: Network Pharmacology-Based Strategy for Predicting Active Ingredients and Potential Targets of Coptis chinensis Franchin Polycystic Ovary Syndrome
Source: Evid Based Complement Alternat Med. 2021 Aug 18;2021:6651307. doi: 10.1155/2021/6651307 (PMC8390140; doi:10.1155/2021/6651307)
Supplement: Supplementary Materials — Figures S1. Molecular docking between the active ingredients of C. chinensis and related targets. Four bioactive ingredients from C. chinensis docking were selected with four targets of PCOS. A, B, C, and D represent the virtual molecular docking results of MAPK1, CLCX8, IL-6, IL-1β, and quercetin, respectively. E, F, G, and H represent the virtual molecular docking results of MAPK1, CLCX8, IL-6, IL-1β, and berberine, respectively. I, J, K, and L represent the virtual molecular docking results of MAPK1, CLCX8, IL-6, IL-1β, and canadine, respectively. M, N, O, and P represent the virtual molecular docking results of MAPK1, CLCX8, IL-6, IL-1β, and berberrubine, respectively. In the network diagram, the light green circle represents the drug (C. chinensis), the green diamond represents the active ingredient contained in C. chinensis, and the green hexagon represents the target, where the active ingredient of the drug is mapped to the target of the disease. [file 6651307.f1.docx]

**Supplementary material**


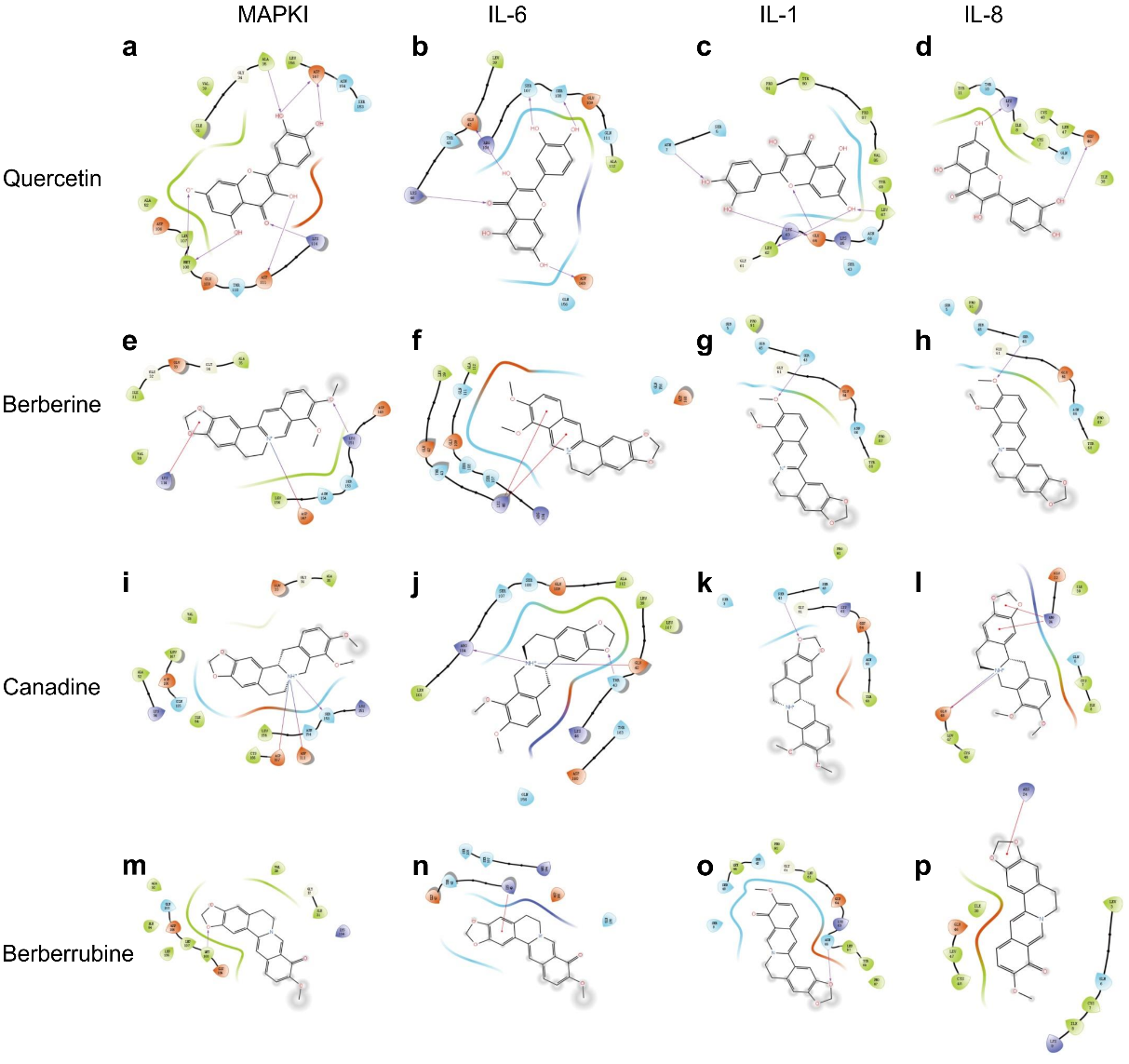


**Figures S1.** Molecular docking between the active ingredients of CC and -related targets. Four bioactive ingredients from CC docking were selected with four targets of PCOS. A, B, C, and D represent the virtual molecular docking results of MAPK1, CLCX8, IL-6, IL-1β, and quercetin, respectively. E, F, G, and H represent the virtual molecular docking results of MAPK1, CLCX8, IL-6, IL-1β, and berberine, respectively. I, J, K, and L represent the virtual molecular docking results of MAPK1, CLCX8, IL-6, IL-1β, and canadine, respectively. M, N, O, and P represent the virtual molecular docking results of MAPK1, CLCX8, IL-6, IL-1β, and berberrubine, respectively. In the network diagram, the light green circle represents the drug (CC), the green diamond represents the active ingredient contained in CC, and the green hexagon represents the target where the active ingredient of the drug is mapped to the target of the disease.
